# Child Health and Mortality

**Published:** 2008-09

**Authors:** Shams El Arifeen

**Affiliations:** ICDDR, B, Mohakhali, Dhaka 1212, Bangladesh

**Keywords:** Child health, Child mortality, Infant health, Infant mortality, Bangladesh

## Abstract

Bangladesh is currently one of the very few countries in the world, which is on target for achieving the Millennium Development Goal (MDG) 4 relating to child mortality. There have been very rapid reductions in mortality, especially in recent years and among children aged over one month. However, this rate of reduction may be difficult to sustain and may impede the achievement of MDG 4. Neonatal deaths now contribute substantially (57%) to overall mortality of children aged less than five years, and reductions in neonatal mortality are difficult to achieve and have been slow in Bangladesh. There are some interesting attributes of the mortality decline in Bangladesh. Mortality has declined faster among girls than among boys, but the poorest have not benefited from the reduction in mortality. There has also been a relative absence of a decline in mortality in urban areas. The age and cause of death pattern of under-five mortality indicate certain interventions that need to be scaled up rapidly and reach high coverage to achieve MDG 4 in Bangladesh. These include skilled attendance at delivery, postnatal care for the newborn, appropriate feeding of the young infant and child, and prevention and management of childhood infections. The latest (2007) Bangladesh Demographic and Health Survey shows that Bangladesh has made sustained and remarkable progress in many areas of child health. More than 80% of children are receiving all vaccines. The use of oral rehydration solution for diarrhoea is high, and the coverage of vitamin A among children aged 9-59 months has been consistently increasing. However, poor quality of care, misperceptions regarding the need for care, and other social barriers contribute to low levels of care-seeking for illnesses of the newborns and children. Improvements in the health system are essential for removing these barriers, as are effective strategies to reach families and communities with targeted messages and information. Finally, there are substantial health-system challenges relating to the design and implementation, at scale, of interventions to reduce neonatal mortality.

## MILLENNIUM DEVELOPMENT GOAL OF REDUCING CHILD MORTALITY IN BANGLADESH

Is Bangladesh on target for achieving the Millennium Development Goal 4 (MDG 4) of reducing child mortality? According to a recent analysis by the United Nations, of the 10 regions covering developing countries, five (North Africa, East Asia, Southeast Asia, Latin America/Caribbean, and the former Soviet Republics in Europe) were on track to achieve MDG 4 (1). South Asia is described as still having high mortality, and the MDG 4 target is not expected to be met by 2015. Sub-Saharan Africa still had very high mortality while the three regions (West Asia, Oceania, and former Soviet Republics in Asia) had moderate mortality and unlikely to meet the MDG 4 target if the current trends prevailed. In a recent analysis of 68 countries of the world, selected because they together account for 97% of maternal, newborn and child deaths worldwide each year, the United Nations Children's Fund (UNICEF) reports that only 16 were on track to achieve MDG 4, including Bangladesh (2).

**Key messages**

- Bangladesh needs to sustain its efforts to achieve MDG 4 of reducing under-five mortality by two-thirds.
  Strengthening and implementing programmes to reduce neonatal deaths and deaths due to ARI is critical.
- *Exclusive breastfeeding rates are not improving at all*.
  Large-scale programmes are needed that provide appropriate counselling and support to pregnant and lactating women.
- There have been significant improvements in child immunization and vitamin A rates, and these need to be sustained.

An earlier joint report in February 2005 by the Government of Bangladesh and the United Nations Country Team in Bangladesh expressed concern at the slowing of the pace in the decline of under-five mortality, based on data available at that time (3). The target for Bangladesh is to reduce under-five mortality from 151 deaths per 1,000 livebirths in 1990 to 50 in 2015. The report estimated that, if this MDG is to be achieved, the country needs to achieve and maintain a reduction of three childhood deaths per 1,000 livebirths every year. The report highlighted the need to focus attention on neonatal and perinatal causes of death, deaths due to pneumonia, diarrhoea, injuries, poor care-seeking practices, malnutrition, and low birthweight (LBW).

What can we say about achieving MDG 4 of reducing child mortality in Bangladesh from the findings of the five Bangladesh Demographic and Health Surveys (BDHS) since 1993 (4). From 1991 to 2004 (mid-years of the BDHS 1993–1994 and 2007), under-five mortality in Bangladesh declined by almost half, i.e. at an average rate of 5.3% per year (Table), which exceeds the required annual decline of 4.3% needed to achieve the MDG of a two-third reduction in under-five mortality by 2015 from the 1990 levels. However, if this estimate is disaggregated, the decline was almost 10% among 1-4 year(s) old children and about 6% annually among post-neonates [1-11 month(s)]. However, the reduction was only 2.6% annually in neonates. Much (57%) of the under-five mortality is now in neonates, and it seems unlikely that the high rates of decline will be sustained among children aged over one month. We actually observed substantial slowing down between 1997 and 2001. It is obvious that, if substantial reductions in neonatal mortality is not achieved, Bangladesh may not achieve MDG 4 (Table 1). While the trends in mortality suggest that we need to ‘work harder’ in achieving MDG 4, it may be that we also need to ‘work smarter’ in identifying the remaining issues that are preventing further improvements.

**Table 1:** Trends in under-five mortality rates in Bangladesh and annual average rates of reduction

	Mortality rates (per 1,000 livebirths)				
Survey>	BDHS 1993–1994	BDHS 1996–1997	BDHS 1999–2000	BDHS 2004	BDHS 2007
Appropriate reference period>	1989–1993	1992–1996	1995–1999	1999–2003	2002–2006
Mid-year>	1991	1994	1997	2001	2004
Under-5 mortality	133	116	94	88	65
Postneonatal mortality [1-11 month(s)]	35	34	24	24	24
Neonatal mortality	52	48	42	41	37
	Annual average rate (%) of reduction in mortality				
	1991–1994	1994–1997	1997–2001	2001–2004	1991–2004
Under-5 mortality	-4.5	-6.8	-1.6	-9.6	-5.4
Child mortality [1-4 year(s)]	-9.5	-6.8	-5.4	-16.4	-9.3
Postneonatal mortality [1-11 month(s)]	-1.0	-11.0	-0.0	-14.5	-6.3
Neonatal mortality	-2.6	-4.6	-0.6	-3.4	-2.6

BDHS=Bangladesh Demographic and Health Survey

## GENDER, URBAN-RURAL AND ECONOMIC DIFFERENTIALS IN CHILD MORTALITY IN BANGLADESH

**Fig. 1:**
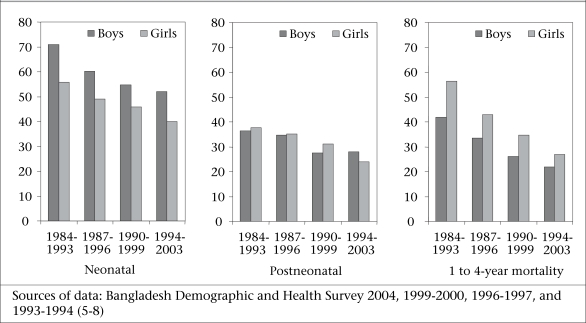
Trends in childhood mortality in Bangladesh (per 1,000 livebirths)

We do not yet have any disaggregated data from the BDHS 2007, which could be included in this assessment. Mortality rates among female children aged 1-59 month(s) have been declining faster than among boys (Fig. 1). The overall under-five mortality rate in the BDHS 2004 (5) is almost 10 points higher for boys than for girls, largely due to much lower neonatal mortality among girls, which is the norm in low-mortality populations. In the age-group of 1-11 month(s), girls are dying less often than boys, which is a major shift from the rates seen in 1993–1994 and 1996–1997 when boys and girls were at par. The mortality gap has been narrowing between boys and girls aged 1-4 year(s), but death rates are still high for girls. These changes likely reflect the changing attitude towards girls and their economic and social values in the Bangladeshi society.

**Fig. 2:**
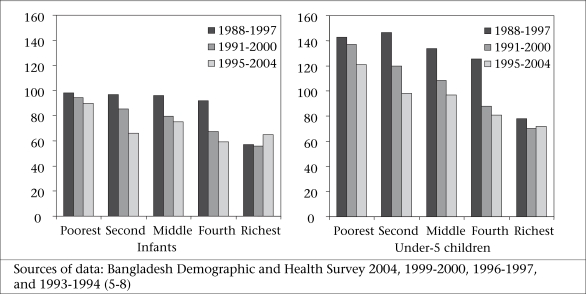
Trends in infant and under-five mortality rates, by wealth quintiles (per 1,000 livebirths)

Figure 2 compares the trends in infant and under-five mortality by wealth quintiles. Since these are based on information up to 10 years prior to the surveys, the periods overlap. However, certain trends are obvious. Gains in infant mortality have been entirely in the three middle quintiles, with almost no change in the poorest or the richest quintile. For under-five mortality, we also notice that the largest gains are in the three middle quintiles with smaller gains in the poorest. The under-five mortality in the richest quintile is static. Although the poorest-richest ratio has been improving (infant mortality rate) from 1.72 to 1.38 and under-five mortality rate from 1.83 to 1.68), the poorest are not getting benefits in terms of reduction in mortality.

**Fig. 3:**
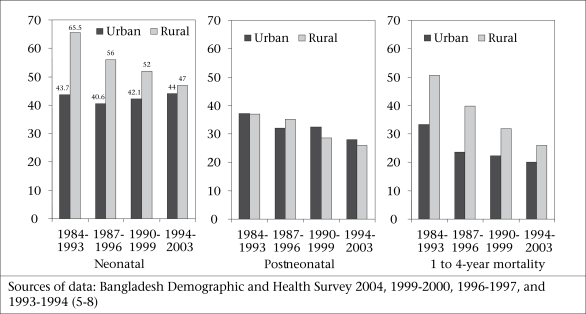
Trends in urban and rural under-five mortality rates (per 1,000 livebirths)

Although there has been a sustained reduction in mortality of neonates and children aged 1-4 year(s) in rural areas (Fig. 3), the absence of a decline in urban areas and the levelling-off of mortality rates at a relatively high level suggest that the decline in rural areas may not be sustained for long. The urban rates may reflect the lowest levels achievable with existing programmes at current coverage levels. This may mean that the recent declines in overall under-five mortality in Bangladesh may not be easy to sustain.

## WHAT NEEDS TO BE DONE TO ACHIEVE MDG 4 IN BANGLADESH

The answers to this question lie, to some extent, in our knowledge about when and why these children die. Recent global reviews indicate that almost 60% of under-five deaths can be prevented by taking known/existing interventions to scale and reach a high coverage of those who need these interventions (9). In Bangladesh, the BDHS showed that, while neonatal deaths contributed only 39% of all under-five deaths in 1991, it is more than half (57%) of under-five deaths in 2004 (4). Birth asphyxia is responsible for a fifth of neonatal deaths while infections, such as sepsis, acute respiratory infection (ARI), and diarrhoea, cause 45% of these deaths (5). Almost two-fifths of neonatal deaths are associated with prematurity of the newborn/LBW. In postneonates, the single biggest killer is ARI, followed by other infections, which together account for about 72% of these deaths. In older children aged 1-4 year(s), we see a similar pattern with a large contribution from ARI and other infections. However, one-fifth of these deaths are due to injuries, particularly drowning. Among all under-five children, prematurity of the newborn/LBW and malnutrition together contribute to about 45% of all deaths.

The age and cause of death patterns indicate certain interventions that need to be scaled up rapidly to reach a high coverage, if one still hopes to achieve the MDG of reducing under-five mortality. These include skilled attendance at delivery, postnatal care for the child, and prevention and management of infections.

### Skilled attendance at delivery and postnatal care

Skilled birth attendants are important because of their critical role in preventing neonatal deaths. Progress in ensuring skilled attendance at delivery has been very limited in Bangladesh, increasing from about 9% in 1993–1994 to 18% in 2007 (4-8). The situation is actually much worse than this since this rate was only 7% and 13% in 1993–1994 and 2007 respectively in rural areas (4). Only 19% of newborns in the 2007 survey received care from a trained provider within two days of birth, and 78% received no care within 42 days postpartum. A majority of neonatal deaths occur in the first few days of birth, and most can be attributed to birth asphyxia, LBW/prematurity of the newborn, and sepsis. It is inconceivable that one can achieve a substantial decline in neonatal mortality if rates of skilled assistance in delivery and postpartum care are not increased. It is reported that, achieving national coverage with skilled birth attendants (SBAs) may require at least 15-20 years, and even then, uncertainties remain as to whether the newly-trained SBA cadre can reach effective coverage. Recent reviews on neonatal health (10) and experiences from India, Nepal, and Bangladesh suggest that other community-based interventions, such as health education to improve neonatal care practices and care-seeking for illness, and creating demand for skilled care, can be used for improving neonatal survival. A policy dialogue and decisions on such interventions seem essential and urgent.

### Prevention and management of ARI

Currently, case management of ARI remains the primary tool for reducing deaths due to ARI in this country. Unfortunately, the percentage of children with ARI taken to a facility or to a health worker has declined in rural areas from 31% in 1996–1997 to 25% in 2007, although the 2007 estimates were a substantial improvement over 17% seen in the BDHS 2004 (4-8). The differences in illness and provider definitions do not make this comparison completely valid; however, the trend does highlight lack of progress and the serious challenges that impede the achievement of a high coverage of ARI case management. This is in sharp contrast to the consistent increase in the use of ORS for the management of diarrhoea, up from 50% in 1993–1994 to 77% in 2007. In the future, vaccines for ARI and zinc therapy will assume a greater role in reducing mortality from ARI (see the paper on infectious diseases in this issue for further discussion).

### Use of ORS in diarrhoea and deaths from diarrhoea

Much has been written about the experience and successes in Bangladesh in taking oral rehydration therapy for diarrhoea to scale. The evidence from the five BDHSs confirms this achievement (4-8). In 1993–1994, 59% of urban children with diarrhoea received ORS while only 49% of rural childhood diarrhoea cases received ORS. In the 1996–1997 survey, we observed a 12-percentage point increase in urban areas, but a decline in rural areas. However, since then, there has been a large increase (to 76% in 2007) in the use of ORS in rural areas, along with small increases in urban areas. In 1995, the Government initiated a major communication campaign on childhood diarrhoea, including its management with ORS. At the same time, the ‘lobon-gur’ solution, widely popularized in the 1980s, was abandoned due to concerns about the lack of standardization of the home-made saline and in view of the wide availability of packaged ORS through health facilities, health workers, and social marketing outlets. In the future, vaccines for rotavirus, cholera, and enterotoxigenic *Escherichia coli* will be increasingly important in reducing the burden of diarrhoeal diseases.

### Breastfeeding

The 2003 *Lancet* series on child survival identified four interventions of established efficacy in preventing deaths due to ARI (9). These include breastfeeding, complementary feeding, *Haemophilus influenzae* (Hib) vaccine, and zinc. Among these, the Hib vaccine will soon become included in the government EPI system, and zinc, now widely available, is recommended for use in each episode of diarrhoea, thereby reducing future episodes of both diarrhoea and pneumonia. Thus, there is optimism that these two new interventions will help reduce deaths due to ARI. Changes in the other two interventions are less likely to improve quickly.

The BDHS showed that the proportion of newborns who started on breastmilk within one hour of birth has increased from 9% in 1993 to 24% in 2004 (data not yet available from the BDHS 2007) (5-8). However, the median duration of breastfeeding has remained unchanged at about 32 months, and there has been absolutely no change in rates of exclusive breastfeeding.

**Fig. 4:**
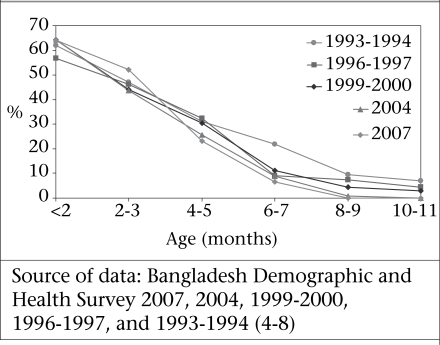
Trends in exclusive breastfeeding in Bangladesh

The consistent and very similar patterns of exclusive breastfeeding over more than 10 years (Fig. 4) show that various efforts in improving the practice of exclusive breastfeeding, including Baby Friendly Initiatives, behaviour change communication (BCC) efforts, etc., have had minimal impact, if any. A critical review of the situation and programmes is essential. There is considerable evidence that, with appropriate counselling and support, more women are able to breastfeed exclusively. These include antenatal education, showing and helping mothers how to breastfeed, and continuing support. As the coverage of antenatal care increases, the opportunities now exist to reach a large proportion of pregnant women with messages on exclusive breastfeeding. However, low levels of skilled birth attendance and postpartum care exert barriers to providing further support and guidance to the newly-delivering woman. Although studies in Bangladesh and elsewhere have demonstrated the value of community-based peer-counsellors in the promotion of breastfeeding, these interventions have not been widely implemented (11). Using the existing community-based government workers is a possibility but this option will need to overcome several challenges (discussed later) before it can be effective.

## PROGRESS IN IMPROVING CHILD INTERVENTION COVERAGE IN BANGLADESH

The BDHS 2007 showed that, in many areas of child health, Bangladesh has made sustained and remarkable progress. The immunization coverage has continued to improve in Bangladesh. The BCG coverage is now 97.3% in urban areas and 96.6% in rural areas. Continuing improvement in rural areas and achievement of parity with urban areas are remarkable. It implies that the outreach programme is now similar in both urban and rural Bangladesh. The coverage of measles vaccination in urban areas is now more than 88%, and it is catching up in rural areas, where the coverage is 82%. The later has been possible as a result of almost 15% increase between 1999–2000 and 2007 (4-8). However, the most remarkable achievement has been in the percentage of 12-23 months old children who have received all vaccines. In urban areas, it is 86% (an increase of almost a fourth from 1999–2000), and in rural areas, it is 81% (more than a third increase from 1999–2000). The goal of immunizing at least 80% of children with all vaccines has been achieved, the challenge now being to sustain it as the programme expands to include other priority vaccines.

The use of ORS can be considered to be a tremendous success for Bangladesh with continuing high use and recognition of its value. Rates of deaths due to diarrhoea have decreased remarkably over the last two decades.

The coverage of vitamin A among children aged 9-59 months has been consistently increasing in every BDHS since the first survey in 1993–1994 (4-8). Much of this achievement can be attributed to linking supplementation of vitamin A to National Immunization Days (NIDs) for polio vaccination. The first NID was held in Bangladesh in 1994. This success demonstrates the advantages of using a campaign-based strategy to deliver interventions that need only to be delivered intermittently. It remains to be seen if this coverage can be sustained, but this programme may be one of the most effective in terms of improving child survival.

## HEALTH SYSTEM CHALLENGES

Poor quality of care, misperceptions regarding need for care, and other social barriers underlie the low level of care-seeking for illnesses of the newborns and children in Bangladesh. There is, however, evidence that, with appropriate training and sustained support, the quality of care from the government first-level facilities can be substantially improved (12). It has also been shown that such improvements in quality of care, along with targetting families and communities with well-designed messages and counselling, can improve the use of these facilities. There are important lessons here that can be scaled up to contribute to a reduction in under-five mortality.

A key lesson here is that good-quality and sustained supervision and support are indispensable. The ongoing Health, Nutrition and Population Sector Programme needs to implement effective strategies in achieving good-quality supervision and support. While various projects in the past have been successful in ensuring adequate supervision and monitoring, this has not been translated into sector-wide systems that function effectively. Many of these successes in the past came when programmes created semi-independent or external supervisory and monitoring systems that did not depend only on managers at different levels who usually are too busy with routine work to be effective supervisors and monitors. Examples are the current EPI and the polio-eradication programme that have successfully used an independent network of monitors.

Financing of supervision is also a barrier. Simply wishing for effective supervision in our sector programmes will not create it. This kind of monitoring requires additional finance to design and support appropriate supervisory systems.

The second key lesson is the need to reach families and communities with targeted messages and information. Families and parents require information to make the right decision regarding care for their children. They often need support to change deeply-ingrained practices. The very large government community-based work force (Health Assistants and Family Welfare Assistants) would seem ideal for reaching the community with such information. However, structural problems, e.g. many of these workers being non-residents in their work areas, and an overwhelming emphasis on providing ‘hard’ services, such as immunizations, contraceptives, etc., are barriers to their assuming this role effectively. Under the current system, they have little incentive to spend their time on communication, even if we were able to provide them with appropriately-designed training on communication, tools, and messages.

There are also health system challenges relating to the design and implementation, at scale, of interventions to reduce neonatal mortality. There is a clear need for community-based programmes, as demonstrated recently from a community-based trial in Sylhet, Bangladesh (13). The Government of Bangladesh is currently developing a national health strategy on the newborns with the expectation that such a strategy will provide guidance and impetus for integrated health programmes on the newborns.

[Note: some of the materials have been previously presented in Bangladesh demographic and health survey 2004 report, chapter 14.]
